# Dr. Wichmann's Observations on the Nettle-Rash Fever

**Published:** 1803-12-01

**Authors:** T. M. Winterbottom


Medical and Phyſical Journal.

VOL. X.] DECEMBER 1, 1803. [NO. LVIII.

*Printed for R. PHILLIPS, by W. Thorne, Red Lion Court, Fleet Street, London.*

### Dr. WICHMANN's OBSERVATIONS *on the* NETTLE-RASH FEVER; *translated from the German, by* T. M. WINTERBOTTOM, M. D.

FEBRIS Urticata differs from Urticaria only in the continuance of the disease and in the fever; for the eruption is precisely the same in both. The fever has slight paroxysms, which however allow the patient to keep out of bed; but is attended with no particular symptom, except an insupportable itching, and a frequent sensation of anxiety, sometimes accompanied with a slight belly-ache. In the chronic state of the disease, the eruption frequently disappears, and after an uncertain period, affects some other part, and in this manner alternates for months. In the febris urticata the eruption is always present and continues several days, but when these are past, it disappears and returns no more. In the febris urticata, the eruption is commonly greater than in the urticaria, spread over a larger surface, and in a violent degree of the disease the face is swoln and disfigured by the confluent bumps or tumours; the hands and feet are also so much tumefied that the patient can scarcely bend his hand. Sometimes, though rarely, a salivation comes on, which with the itching and burning eruption is very troublesome. The utmost limits of the fever are nine days, and it yields to such mild remedies, that I shall no longer dwell upon it, but proceed to consider the disease in its chronic state.

In some, the eruption continues without any fever only for a few days, in others for an indeterminate time of weeks, months, and even years; that is, it frequently disappears during this long period for several days; again returns, and again disappears, but the disposition to the disease still continues. In some subjects, during the whole time, some of the eruption is visible on the skin. One child teased me for two entire years, and another exhausted my patience for nearly four years. Heberden has seen it continue for two years in different patients, with very short intervals, and in some from seven to ten years. (Medical Transact. vol. ii. p. 175.)

The name of this disease, like that of Pemphigus, is very characteristic in several languages; and when mistaken, it arises from the skin not always assuming the same appearance as when stung by a nettle, insect, &c. For it is still urticaria, though very different in appearance from the sting of a nettle or the bite of an insect: 1. When long, red, hard stripes, like the stroke of a whip, appear on the skin. 2. When the whole surface of the back appears of a brown red, and the painful itching alone distinguishes it from scarlet fever. 3. When the bumps in the face run so much together, as to cause the face to swell, and bear no resemblance to the insulated discrete appearance excited by the bites of insects. Another appearance in which the eruption nearly resembles itch, will afford an opportunity of treating it more fully.

The urticaria, whether acute or chronic, is visibly elevated above the skin, is hard, firm, solid, more of a dead pale colour than red; in the centre it is almost white; contains no fluid, and is neither elevated into a point (or stigma), nor covered with a crust; it likewise never suppurates. This is not perfectly exact in the bite of an insect, where the bump or tumour is frequently not so smooth and level, but has a small elevation. The usual circumference, when the papulæ stand single, is seldom larger than the nail of a finger, and often less; but when they touch, the skin is raised, for the size of the hand, and they produce in the part such a degree of swelling, that they can no longer be recognized for papulæ, (what the English call wheals) but the whole figure of them becomes very irregular and undescribable. Perhaps my readers, who have not seen them, may understand me better if I say in another language, " Les tubercules forment (says Lieutaud in his Precis de la Medic. pr.) de larges plaques relevées avec ardeur & demangeaison."

If the *phymata* appear insulated, they have generally a very red circumference, but disappear in a short time, and without any particular cause appear again. They are confined to no particular part, but break out any where on the surface.

The greatest degree of the disease is seen when it appears in consequence of having eating muscles (mytelus edulis); edulis); for, with regard to the identity of the eruption which appears from this cause, and that just described which arises from no evident cause, no doubt can be entertained. The disease arising from this cause, which has been accurately described by Berens, Werlhof, Mohring, &c. is, as well as the other, more frightful than dangerous, and is usually quicker in its course than the real febris urticata; being frequently over within twelve hours, (so that it seems inexplicable how Kock, p. 13, can suppose the chronic disease to arise from it). Except great anxiety, patients chiefly suffer from the intolerable itching; and the eruption sometimes becomes so confluent, especially in the face, that the eyes are nearly closed and the face disfigured by the swelling.

Since muscles have not been brought so abundantly to Hanover, where they are looked upon as delicacies, and brought from the East Sea by way of Kiel, physicians have lost the opportunity of observing the disease, and I cannot recollect when I saw a similar instance. In 1793 I saw a violent attack of the disease in a man, which arose from no evident cause, and which did not terminate till the ninth day. The violent burning and itching were united with fever and a salivation of three days continuance; this last has been remarked by Professor Kock, who refers it to the eruption affecting the inside of the mouth, and irritating the salivary glands.

The cause of this poisoning by muscles has lately become better understood in the Netherlands, where these muscles, particularly on the banks of the Scheld, are abundantly used; and the opinion formerly entertained by Werlhof, has been corrobated, that the muscle itself does not contain the poison. See Memoires de l'Acad. des Sciences & Belles Lettres de Bruxelles, 1771, tom. i. p. 229, and tom. ii.

One of my acquaintances here invariably suffers a slight degree of the disease, sometimes accompanied with head ach and fever, every time that he eats the common river cray fish, (astacus) an observation which has been long made, and lately by Gruner, Rode, &c.; by Sauvages it was observed from eating of the liver of a fish; and by others, from eating other things.

Whether muscles, crabs, &c. can throw any light upon this disease, must be determined by pathologists; it has nothing to do with diagnostics. The pain in the belly, which sometimes occurs in urticaria, together with the effects of muscles, &c. point out to the physician that it is

 not not a local disease of the skin, neither is much use to be expected from diaphoretics, baths, &c.

It is one of the peculiarities of this disease, that it may not only be ranked among the impetigines and also the exanthemata, as I hope will appear from what has been said, but when in the chronic state a great susceptibility for this disease once prevails, the disposition to it is so strong in the skin, that it may often be produced at pleasure, by merely pressing upon the arm with the finger and carrying it downwards; the course of the finger is marked by a hard redness, which remains for some time visible. It appears to me not less remarkable that the eruption commonly disppears in bed, and again appears on exposure to the cold air; a phenomenon certainly not observed in other eruptive diseases, and which seems to form a characteristic of this one.

It may be further noticed as somewhat singular, that in 1769, I saw a child, two years old, in which the eruption almost resembled tugillations, they were blue, and here and there of a blackish hue; but in a few days they disappeared by simple remedies, and were not attended with fever. I have also seen this eruption in a high degree in a woman, complicated with tertian fever: the eruption always appeared on the day of attack, and disappeared with the fever. It was at length cured, together with the tertian, by the use of the bark alone. In a child five years of age, I found this eruption on the third day after the appearance of the benign small-pox; in a short time the urticaria disappeared, without affecting the course of the principal disease, or producing any uncommon symptoms.

Did the chronic eruption urticaria always possess the the same characteristic form and circumscribed figure as has been described, it would be foolish, at least very useless, to compare it with itch. The reader will not, it is presumed, expect as exact a description of itch as has been given of urticaria, and I presume it is well known by the constant spherical figure of its pustules. But what induces me to compare them here, (and I cannot too much excite the reader's attention to it) is, that both these complaints have often the most striking resemblance to each other, and then deceive the most experienced eye. In long continued and frequently returning urticaria, the patient, especially children, cannot abstain from scratching, but break through the bumps, and make little wounds; these often cause the eruption to lose its original form and assume the appearance of small papulæ containing a purulent lent fluid; or there arises a small *(Button, Knopfchen)* in the midst of it, which resembles a dry itch pustule, but not a perfectly recent one, especially when it takes in a large surface of the skin; and is more apt to mislead, as the itching continues from time to time as in the itch, and these little tubercles *(knopfchen)* do not disappear as in nettle rash. In the first instance of this kind, in a young child, I was perplexed for half a year, and did not discover my error until I accidentally observed the first appearance of the eruption, and discovered evident, broad papulæ, which afterwards changed as already described. The conviction that the child during a long period had infected no one, and that no person in the house had laboured under any suspicious eruption, or any similar one to the present, rendered me more certain respecting the nature of this eruption.

I find that this appearance has not escaped the accurate Heberden; but many others who do not observe the disease long enough, or at different periods, overlook the difference between the real itch and this eruption, and even attempt to remove it by external remedies; which produces the mischief so often ascribed to repelled itch. I shall therefore here point out wherein the distinction and similarity between the two diseases exist.

1. The first appearance of both eruptions immediately decides the difference, and any one who can observe them at this early period cannot easily get wrong; for the itch, as soon as it is actually visible, has well defined, hard papulæ, which may be felt above the skin, and which continue to grow larger; retain the same spherical, or rather hemispherical form, and slowly form pustules; not so urticaria.

2. The itch is therefore a pustular disease; i. e. a certain time after its origin and visible formation upon the skin, it contains a fluid. Urticaria forms phymata, i. e. hard, solid, rather flat elevations, which never contain a fluid; are never round, but of an irregular figure, and distinguish themselves from the itch pustule by their greater circumference, both in their origin and progress.

3. The uticaria is in full vigour from the first hour of its appearance; an itch pustule is much slower, often for days or weeks, until several of them are spread over the skin.

4. The itch, after a long continuance, forms small scabs, or crusts, from inspissated moisture or pus; urticaria never; for though we sometimes see upon their papulæ little

 knopfchen, knopschen, yet upon a nearer inspection they will be found to have arisen from scratching.

5. Itch speedily infects; urticaria, after even the longest continuance, never.

6. The itch, in itself, is always a chronic disease, never excites, as sometimes does urticaria, fever, nor is it a symptom of fever.

7. Itch spares the face; urticaria no part of the body, and least of all the face.

8. Scabies itches most in the heat of bed, less in the open air, and not at all in a severe cold, (which is explained by the animattion or benumbing of the itch animalcules). Urticaria generally itches most in the open air, and often more in a cold air; in the warmth of bed the erruption either disappears, or at least itches less.

9. In urticaria no external remedies are effectual, neither quicksilver nor sulphur, both of which soon show their specific effects in itch, as being merely a disease of the skin; and whosoever does not speedily obtain the wished for effects, in a case of supposed itch, must have mistaken the complaint, or should suspect a complication of some other disease with it, or even the nettle rash, which is not solely a disease of the skin.

Both these diseases resemble each other, in having the eruption visibly (and palpably) elevated above the skin, with violent itching; and though both complaints be of long standing, they are unattended with fever; for the true urticaria never appears towards the conclusion of a chronic eruption; but where it is seen, it has been present from the first appearance of the eruption.

The difference between both is so great and evident, that they required to be compared merely from the circumstances above mentioned. There are certainly exanthemata which bear a closer resemblance to the itch than urticaria, but I shall pass them over at present. It is much more difficult to distinguish measles from the nettle eruption: when combined with fever, it does not form urticaria, which disappears for some time and again appears, but consists in an acute disease, which continues visible upon the skin for several days. The mistake is more easy when a case of urticaria occurs in a place where measles prevail at the same time. I have observed such an ambiguous instance in a child, where three persons had just recovered from measles in the same house: the absence of cough and other catarrhal symptoms, and the difference of the eruption eruption to the touch, determined me to consider it as urticaria, rather than measles.

Measles and febris urticata agree in the following respects:

1. Both of them form an elevated eruption of an irregular figure, affecting all parts of the body, particularly the face; and,

2. Almost limited to the same period of time.

3. The measles consist also of solid tubercles, spots elevated above the skin, which contain no fluid, and have no crusts upon their summits; and although Wedekind, who has described this eruption most accurately, discovered in many a clear yellowish fluid, yet we ought not to call the eruption pustulous, as this supposed fluid disappears imperceptibly, and does not increase as in itch, small-pox, &c. nor change into pus, and form crusts; hence no pustular disease is produced. Probably the fluid is produced here, in the same way as has been observed in urticaria, by the eruptions having been scratched and broken.

4. The eruption in both complaints is almost equally elevated above the skin, and becomes gradually broader. I know no exanthema which has spots of the breadth and circumference which the measles sometimes, and urticaria have. The spots are usually discrete, insulated; but frequently they touch and become maculæ corymbosæ, which the English term '*clustered spots*.'

5. Both complaints occur at all times of the year; and from the number of extensive epidemics which I have witnessed, it is false that the measles are confined to the harvest or winter. It is equally as erroneous, that urticaria appears only in certain months.

Their most striking differences consist in the following:

1. Measles are very infectious, although, indeed, many remain free from them, as well as the small-pox, during their lives; others again escape during one epidemic, and are affected by a succeeding one. The first mark of infection commonly shews itself on the eighth day, or within fourteen days after exposure to a person ill of the disease. Urticaria does not infect; but this mark can be of no use at the beginning of the disease, and in a solitary instance, it is useful only where doubts have occurred.

2. The symptoms are very different even at the commencement of the disease. The measles commonly begin with catarrhal symptoms, running of the eyes and nose; cough, which becomes more violent, and is absent only in very rare instances; oppression or similar disorders of the breast; bleeding at the nose, and dullness of the eyes, which are very sensible of light. In urticaria none of these, particularly the cough and bleeding of the nose, the almost inseparable attendants of measles, are to be observed. The eye-lids are indeed often swoln, but the eye almost never suffers, and does not appear red.

3. In measles, the (morbific) matter determined to the surface of the body is often evolved so slowly, that the catarrhal symptoms have occurred several days (I know an instance of eight days) before the eruption is visible; it is particularly delayed by copious bleeding at the nose; and it is frequently visible on the skin within twenty-four hours of the attack. In febris urticaria, the eruption is commonly earlier than the fever, or at least is always visible in the first twelve hours.

4. The eruptions also differ on close inspection. In measles the spots are red and slightly elevated above the skin; in urticaria they are of a pale red. In the former, a small, almost imperceptible branny punctum gradually forms towards the end of the fever; a kind of slight desquamation takes place. In urticaria the eruption completely disappears, without desquamation or formation of pustules, crusts, &c. In general, we ought to detect the difference, to feel the eruption as well as see it, or try it with the finger more than with the microscope. By this means we discover in urticaria a much more circumscribed hardness, and a greater elevation above the skin; the islands, if, to make it more intelligible, I may use this coarse comparison, have in urticaria a steeper shore, in measles a flatter one. In the former they resemble a rugged granite rock, in the latter a gradually rising shore. To speak in the language of physicians, they stand in the same relation to each other as phlegmone to erysipelas.

5. The painful burning and itching in urticaria is totally wanting in measles.

6. When the urticaria disappears, the whole disease, fever, &c. is removed; and if any doubts have been entertained, they may now be removed. In measles the consequences are often worse than the disease; and are particularly dangerous to the eyes and lungs.

7. The face never swells so much in measles, and the whole skin is never so much puffed up as in a great degree of urticaria, fievre ortiée.

8. Urticaria spares no part of the skin; but measles in general leaves the harder skin of the soles of the feet and palms of the hands free.

9. The

9. The measles are indeed often seen sporadic, but generally they are epidemic in a place; I know of no instance of urticaria being so, from my own experience or reading, and I doubt whether others have ever observed it epidemic.

*South Shields, Nov.* 5, 1803.

